# The promising role of *PAX1* (aliases: *HUP48*,* OFC2*) gene methylation in cancer screening

**DOI:** 10.1002/mgg3.506

**Published:** 2019-01-12

**Authors:** Chao Fang, Sai‐Ying Wang, Yu‐Ligh Liou, Ming‐Hua Chen, Wen Ouyang, Kai‐Ming Duan

**Affiliations:** ^1^ Department of Anesthesiology The Third Xiangya Hospital of Central South University Changsha China; ^2^ Postdoctoral Research Workstation of Clinical Medicine The Third Xiangya Hospital, Central South University Changsha China; ^3^ Xiangya Medical Laboratory Central South University Changsha China

**Keywords:** cancer screening, cervical cancer, human papillomavirus, methylation, *PAX1*

## Abstract

**Background:**

*Paired‐box gene 1* (*PAX1*), a member of the PAX family, plays a role in pattern formation during embryogenesis, and might be essential for development of the vertebral column.

**Methods:**

*PAX1* is silenced by methylation in several cancers and is considered a tumor suppressor gene. Our previous studies reported *PAX1* as hypermethylated in cervical cancer tissues, thereby suggesting it as a potential screening marker. Recently, an increasing number of studies have confirmed *PAX1* methylation as a promising biomarker in cervical cancer based on its excellent discriminatory ability between high‐grade cervical lesions and normal tissues, resulting in a reduced necessity for referral for colposcopy and biopsy. Additionally, *PAX1* is also hypermethylated in other tumors, including those associated with epithelial ovarian cancer, esophageal squamous cell carcinoma, head and neck squamous cell carcinoma, and endometrial carcinoma, and shows relatively good sensitivity and specificity for the detection of these tumors.

**Results:**

This review summarizes reports of *PAX1* methylation and its promising role in cancer screening, especially that associated with cervical cancer.

**Conclusion:**

According to current evidence, combined testing for human papillomavirus and *PAX1* methylation analysis represents an efficacious cervical cancer‐screening protocol.

## 1 INTRODUCTION

*PAX1* (OMIM: 167,411) gene, a member of the PAX family located on chromosome 20p11.2, is essential to regulate fetal development*.* (Schnittger et al., 1992). Members of the PAX family typically contain a paired‐box domain and a paired‐type homeodomain, which are essential during fetal development and play critical roles during development of the vertebral column (Wallin et al., 1994). *PAX1* plays a role in sclerotome differentiation and interacts with homeobox (*HOX*) genes, which play a prominent role in normal fetal development and controlling cell proliferation (Cillo, Cantile, Faiella, & Boncinelli, 2001). Substitution or deletion of this gene in mice produces variants associated with vertebral malformations and Klippel–Feil syndrome (Hol et al., 1996; McGaughran, Oates, Donnai, Read, & Tassabehji, 2003). Methylation of *PAX1* promoter is an important epigenetic regulation associating to the development and the metastasis of the tumor. *PAX1* gene in cervical and ovarian cancer is silenced by methylation and is considered as a tumor suppressor gene (Chang et al., 2014; Hassan, Hafez, Kamel, & Zekri, 2017; Kan et al., 2014; Kong, Du, Wang, Yang, & Zhang, 2015; Su et al., 2009).

## 2 *PAX1* METHYLATION AND CERVICAL CANCER

### 2.1 Challenges in the diagnosis and treatment of cervical cancer

Cervical cancer is the second most common carcinoma among women worldwide (Torre et al., 2015) and has a long pre‐invasive phase. During cervical cancer development, normal cervical cells gradually develop into precancerous lesions; however, cervical cancer can also evolve from pre‐existing noninvasive premalignant lesions referred to as cervical intraepithelial neoplasias (CINs) that range in severity from CIN1 (mild dysplasia) to CIN2/3 (moderate/severe dysplasia/carcinoma) and which can be maintained over several years (Fabrizii, Moinfar, Jelinek, Karperien, & Ahammer, 2014; Rakotomahenina, Garrigue, Marty, & Brun, 2014). Cervical cancer has a well‐defined CIN process and can be identified and treated before malignancy formation (Jones, 2010; Wentzensen et al., 2013). Given that a long developmental process from each stage of CIN to cervical cancer, early diagnosis and treatment of CIN can effectively prevent cancer from happening.

Infection with human papillomavirus (HPV) represents a primary risk factor leading to cervical cancer (Bosch, Lorincz, Munoz, Meijer, & Shah, 2002; de Silva, Mendis, & Perera, 1999; Helmerhorst, 2000; Kaufman, Adam, Icenogle, Lawson, et al., 1997; Nessa, Rashid, E‐Ferdous, & Chowdhury, 2013; Schiffman & Castle, 2003; Wentzensen et al., 2013; Zielinski et al., 2001). HPV test is the most common screening method for cervical cancer for it high sensitivity; however, HPV test is not recommended for screening purposes because of its low specificity. Moreover, low positive‐predictive values of HPV‐positive testing results have been obtained, even in the presence of clinically relevant lesions along with Papanicolaou (Pap) smear and ThinPrep cytology tests (Cox et al., 1995; Cuzick, 2010; Dane, Batmaz, Dane, & Cetin, 2009; Kaufman, Adam, Icenogle, & Reeves, 1997; Nessa et al., 2013). Additionally, most HPV infections are subclinical, transient, and noncancerous. Evidence suggests that only persistent HPV infections are associated with precancerous lesions, as a positive HPV result might lead to overinterpretation of minor cellular abnormalities, redundant anxiety, and additional testing (Tjalma & Depuydt, 2014), which limit HPV testing as a diagnostic factor for cervical cancer. Therefore, identification of novel and accurate biomarkers for cervical cancer screening remains necessary.

For diagnosis of cervical cancer, suspicious cervical lesions will be initially evaluated by colposcopy in clinical practice, and, if necessary, biopsy samples will be taken for further histopathologic examination (Massad et al., 2013). However, it remains a challenge to choose personalized treatments and follow‐up strategies for biopsy confirmed patients with CINs. Because most CIN1 patients will regress to normal without intervention, and even high‐grade lesions (CIN2/3) exhibit a substantial rate of regression, only a small percentage of dysplasia progresses (Jones, 2010; McCredie et al., 2008; Wentzensen et al., 2013). For patients with naturally regressing CINs, unnecessary surgery can cause adverse effects, such cervical dysfunction, which can result in recurrent spontaneous abortion during subsequent pregnancies (Bjorge, Skare, Bjorge, Trope, & Lonnberg, 2016; Jakobsson & Bruinsma, 2008; Song, Seong, & Kim, 2016), whereas for patients with CINs destined to progress, medical treatment, and follow‐up are needed to prevent cervical malignancy. Therefore, reliable biomarkers are needed to assess the risk of CIN progression, reduce unnecessary referral for colposcopy and biopsy, and avoid overtreatment of patients desiring to preserve fertility.

### 2.2 Relationships between *PAX1* methylation and cervical cancer screening

Epigenetic studies demonstrate that DNA methylation could be a symbolic event of carcinogenesis. Several kinds of DNA methylation are reported as strongly associated with CINs and cervical cancer, including those in *sex‐determining region Y‐box 1* (*SOX1*; OMIM: 602148), *PAX1, LIM homeobox transcription factor 1A* (OMIM: 600298), *NK6 transcription factor‐related locus 1* (OMIM: 602563), and *Wilms tumor 1* (OMIM: 607102) (Chang et al., 2014; Lai et al., 2008; Lim et al., 2010; Lorincz, 2016; Vasiljevic, Scibior‐Bentkowska, Brentnall, Cuzick, & Lorincz, 2014). Among these genes, multiple studies confirmed *PAX1* methylation as the most highly correlated with CIN progression and cervical carcinogenesis (Chang et al., 2014; Chao et al., 2013; Chen et al., 2016; Huang et al., 2010; Kan et al., 2014; Lai et al., 2010, 2014; Luan et al., 2017; Tian et al., 2017; Xu et al., 2015).

In 2008, Lai et al. (2008) first reported that *PAX1* was abnormally methylated in association with cervical cancer, and the *PAX1* gene was silenced by hyper methylation and low expressed in these biopsies of cervical cancer (Lai et al., 2008). Several studies found that *PAX1* methylation increased along with increased disease grade in the following order: *PAX1* methylation in squamous cell carcinoma (SCC) > high‐grade squamous intraepithelial lesion (HSIL) > low‐grade squamous intraepithelial lesion (LSIL) > normal tissue (Lai et al., 2008; Lim et al., 2010; Xu et al., 2015). Detection of high‐grade cervical lesions in patients with atypical squamous cells of undetermined significance (ASCUS) remains a challenge in the screening and diagnosis of cervical cancer.* PAX1* methylation demonstrated better performance as a marker than results of a high‐risk HPV‐DNA test for the detection of high‐grade lesions (CIN2+) in ASCUS cases; however, *PAX1* methylation allows for the screening out of a majority of low‐grade ASCUS cases (Li et al., 2015; Wang, 2014). A result from 443 cervical scraping samples showed that* PAX1* detection alone had a sensitivity and specificity of 86% and 85%, respectively, for the detection of CIN3+ lesions, whereas when used as a co‐test with the Pap test, the sensitivity and specificity were 89% and 83%, respectively (Kan et al., 2014). Additionally, our previous studies found a significant association between methylated *PAX1* and CIN3+ or worse in combination with HPV16/18, with sensitivities and specificities of methylated *PAX1* with HPV16/18 for CIN3+ detection at 89.2% and 76.0%, respectively (Liou et al., 2016), whereas dual methylation testing for *PAX1/zinc protein finger 582* (OMIM: 615600) combined with HPV‐16/18 genotyping resulted in 100% identification of carcinoma in situ or SCC (Tian et al., 2017). Meta‐analyses also supported the utility of *PAX1* methylation as an auxiliary biomarker in cervical cancer screening. One meta‐analysis reviewed 1,385 subjects with various stages of CIN and normal cervical pathology, finding that the sensitivity and specificity of *PAX1* methylation in CIN3+ vs. normal samples were 0.77 and 0.92, respectively (Nikolaidis et al., 2015). Additionally, 15 individual studies showed that single *PAX1* methylation allowed the accurate differential diagnosis of cervical cancer/HSIL patients from normal individuals with a sensitivity of 0.80 and a specificity of 0.89 (Kong et al., 2015).

**Figure 1:**
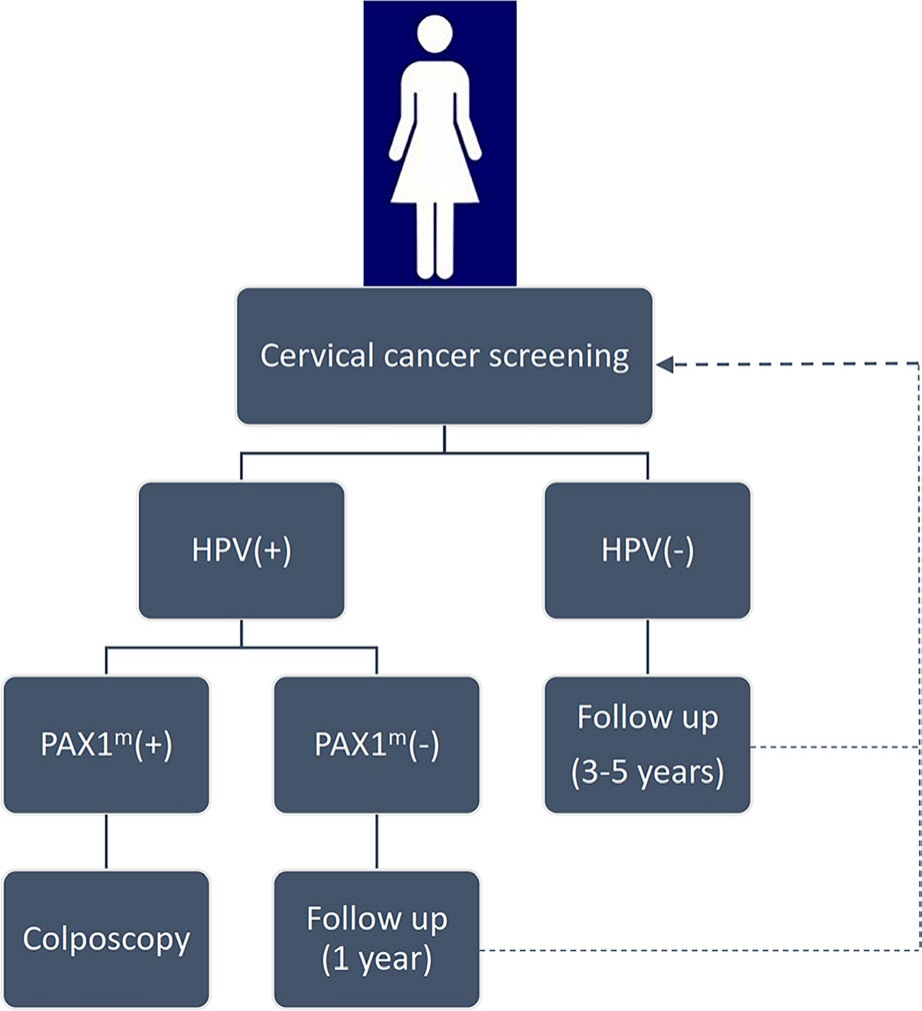
Combined HPV testing and *PAX1*‐methylation detection as a cervical cancer‐screening protocol. During cervical cancer screening for women, HPV‐positive results accompanied by *PAX1*‐methylation analysis allow accurate determination of the necessity for biopsy during colposcopy during diagnosis of CIN3+ lesions. (*PAX1*: NCBI Refseq: NM_006192.4, GRCh38)

These data suggested the efficacy of *PAX1* methylation as a biomarker for cervical cancer screening, and that it plays a guiding role in triage management of LSIL, HSIL, and SCC patients, as well as displays higher accuracy than single HPV‐DNA testing. These findings suggest that incorporating *PAX1‐*methylation detection into current cervical cancer‐screening protocols (Figure 1) will promote the accurate screening of women requiring treatment, reduce unnecessary referrals for colposcopy and biopsy, and ease the burden on patients and medical resources.

## 3 *PAX1* METHYLATION IN OTHER TUMORS

Aside from cervical cancer, *PAX1* also displays hypermethylation in other tumors and offers great promise as a marker for cancer detection (Table 1). Detection of *PAX1*‐methylation levels in oral scrapings or oral swabs indicated that *PAX1*‐methylation levels and positive rates increased along with disease severity (SCC > precancerous lesions > normal oral mucosa) (Cheng et al., 2017, 2016; Huang et al., 2014), but decreased following cancer excision. However, these levels increased again at subsequent sites of recurrence in some cases at ~3‐ to ~4‐months prior to recurrence (Cheng et al., 2016). These studies suggest *PAX1* methylation as an effective biomarker for oral cancer detection and the prediction of oral cancer recurrence.

**Table 1:** *PAX1* *#* gene methylation is a promising biomarker for cancer screening

Cancer	Samples	*PAX1* gene methylation	Clinical application
Cervical cancer	Cervical exfoliated cells	*PAX1* hypermethylation in cervical high‐grade lesions (CIN3+and worse)	Biomarker for Cancer screening; Patients triage management
Oral cancer	Oral scrapings/oral swabs	*PAX1* hypermethylation in tumor samples, methylation levels decreased after cancer excision and increased again 3–4 months before cancer recurrence	Biomarker for Cancer screening; Recurrence prediction.
Esophageal squamous cell carcinoma	Tissues	*PAX1* hypermethylation in tumor tissues	Biomarker for cancer detection
Colorectal cancer	Tissues	*PAX1* hypermethylation in tumor tissues	Biomarker for cancer detection
Head and neck squamous cell carcinoma	tissues	*PAX1* hypermethylation in tumor tissues	Biomarker for cancer detection
Epithelial ovarian cancer	Tissues	*PAX1* hypermethylation in HPV16/18‐infected EOC tissues	/

a NCBI Refseq: NM_006192.4, GRCh38.

In epithelial ovarian cancer (EOC), *PAX1* was significantly hypermethylated in HPV16/18‐infected EOC tissues (Hassan et al., 2017). Another study showed that levels of *PAX1* methylation were significantly higher in esophageal squamous cell carcinoma (Huang, Wang, et al., 2017), colorectal cancer (Huang, Tan, et al., 2017), and head and neck squamous cell carcinoma (Guerrero‐Preston et al., 2014), and the *PAX1* protein levels were lower in endometrial carcinoma (Liu et al., 2016). Moreover, detection of *PAX1* methylation displayed relatively good sensitivity and specificity for the diagnosis of these tumors and holds great promise for tumor screening or as a prognostic marker.

## 4 POTENTIAL MECHANISMS OF *PAX1* METHYLATION

In cancer development, hypermethylation of promoter regions containing CpG islands can inactivate tumor suppressor genes, thereby affecting genes associated with the cell cycle, DNA repair, cell–cell interactions, apoptosis, and angiogenesis (Herman & Baylin, 2003). In most vertebrates, *PAX1* and* PAX9* exhibit similar expression patterns and functions that belong to a highly conserved family of *PAX* genes, encode highly conserved transcription factors, and play roles in pattern formation during vertebrate embryogenesis (Paixao‐Cortes, Salzano, & Bortolini, 2015). Studies show that *PAX* genes promote cell proliferation, cell‐lineage specification, migration, and survival, and roles in tissue development and cellular differentiation in embryos (Dahl, Koseki, & Balling, 1997). In most cases, as fetal development progresses, *PAX* expression attenuates; however, in some tissues, *PAX* expression either persists into adult life or increases to exert functions, such as protection against stress‐induced cell death (Cai et al., 2005). Studies *PAX1* and *PAX9* mutants show that *PAX1* can compensate for the loss of *PAX9*, although not *vice versa* (Wilm, Dahl, Peters, Balling, & Imai, 1998). Little is known about the functional role of *PAX1* in cancer biology. Liu et al. (2016) identified *PAX1* protein levels as potential histopathology biomarkers for the differential diagnosis of malignant and premalignant endometrial lesions (Liu et al., 2016). Another study reported *PAX1* expression‐inducing tumor formation following subcutaneous injection of cultured cells expressing *PAX1* into nude mice (Maulbecker & Gruss, 1993). Additionally, other studies showed that methylation of CpG islands in the *PAX1* promoter region regulates cervical neoplasia (Chen et al., 2016; Kan et al., 2014), resulting in their characterization of *PAX1* as a tumor suppressor gene. Although *PAX1* functions have been hypothesized based on phenotypic outcomes associated with knockout models, *PAX1* molecular functions and target genes remain largely unknown. Using mutation mouse models, studies have identified that *PAX1* regulates epithelial cell death and proliferation during thymus and parathyroid organogenesis (Su, Ellis, Napier, Lee, & Manley, 2001). Studies investigating *PAX1* reactivation in cervical cancer cell lines suggest that this can occur through curcumin and resveratrol administration through their effect on histone deacetylase accompanied by the downregulation of ubiquitin‐like with PHD and RING finger domains 1 (OMIM: 607990), which regulates both DNA methylation and histone acetylation (Parashar & Capalash, 2016). Moreover, *PAX1* methylation levels decrease along with increases in its mRNA expression after silencing of DNA methyltransferase 1 (*DNMT1*; OMIM: 126375), which plays a significant role in maintaining DNA methylation status and regulating the expression of tumor suppressor genes (Zhang et al., 2011). Similar results showed that curcumin, resveratrol, and *DNMT1* influence *PAX1* activity and might represent effective targets for treatment of cervical cancer (Parashar, Parashar, & Capalash, 2017). *PAX1* methylation is also associated with *NOTCH1* mutation and the Hedgehog pathway, which is regulated by HOX transcription factors and enhancer of split 1 (OMIM: 139605) (Bolos, Grego‐Bessa, & de la Pompa, 2007; Forastiere, Koch, Trotti, & Sidransky, 2001; Guerrero‐Preston et al., 2014; Koop et al., 2010; Landsman, Parent, & Hebrok, 2011; Mammucari et al., 2005; Manley & Capecchi, 1995; Mill et al., 2003; Sang, Roberts, & Coller, 2010; Schubert et al., 2005; Wall et al., 2009). Loss of *ΝOTCH1* function due to mutation or the methylation‐dependent silencing of downstream genes, such as *PAX1*, likely abrogates normal cell differentiation (Guerrero‐Preston et al., 2014).

## 5 DISCUSSION AND PROSPECTS

*PAX* genes encode a family of nine transcription factors that act as cell‐lineage‐specific regulators of the tissues where they are normally expressed and are now recognized as important factors in cancer progression. Additionally, these factors might play previously unrecognized fundamental roles in balancing proliferation and differentiation signals. Numerous studies have demonstrated that *PAX1* methylation plays an important role in the progression of cancers and contributes significantly to the sensitivity and specificity of cancer screening, especially for cervical cancer. In scrapings for cervical cancer, analyses indicated that *PAX1* is silenced by hypermethylation. Moreover, *PAX1* methylation plays a guiding role in the triage management of normal tissues, as well as CIN2, CIN3, LSIL, HSIL, and SCC patients. Although HPV testing is appealing for cervical cancer diagnosis, it cannot distinguish whether or not an HPV‐positive result is associated with a clinically relevant lesion. Furthermore, these test results can be subject to overinterpretation and causing unnecessary panic. *PAX1* methylation represents a novel biomarker that exhibits increased specific and accuracy for cervical cancer screening and diagnosis. There is increasing evidence that testing for methylated genes can replace cytology as a reflex test for HPV‐positive women, and interim clinical guidance approves the use of such tests as an appropriate triage tool for HPV (Huh et al., 2015; Luttmer et al., 2016).

Here, we propose a screening strategy for cervical cancer that combines using the HPV testing and *PAX1*‐methylation analysis as triage tests according to current evidence. In HPV‐positive patients, the detection of *PAX1* methylation is necessary for diagnosis of CIN3+ lesions (Figure 1) and will greatly benefit accurate cervical cancer screening, identify women that require treatment, and reduce unnecessary referrals for colposcopy and biopsy. However, additional standardization and large‐scale clinical studies are needed to evaluate the efficacy of *PAX1* methylation for cervical cancer screening and early detection. Additionally, further studies targeting the specific mechanisms associated with methylation‐induced alterations in cellular activity are required to provide additional evidence supporting the clinical use of *PAX1* methylation as a screening tool.

## CONFLICTS OF INTEREST

The authors declare no competing financial interests.
